# A high-efficiency hydrocyclone designed by response surface methodology for acid hydrolysis residue recycling

**DOI:** 10.1098/rsos.172339

**Published:** 2019-01-09

**Authors:** Yanxia Xu, Bo Tang, Xingfu Song, Jianguo Yu

**Affiliations:** National Engineering Research Centre for Integrated Utilization of Salt Lake Resource, School of Resources and Environmental Engineering, East China University of Science and Technology, Shanghai 200237, People's Republic of China

**Keywords:** hydrocyclone, acid hydrolysis residue, classification sharpness, response surface methodology

## Abstract

A high-efficiency hydrocyclone was designed by response surface methodology to evaluate the recycling of acid hydrolysis residues from titanium dioxide (TiO_2_) production as a study case. TiO_2_ is an important product and the world's best white pigment. During its production from ilmenite (FeTiO_3_) by the sulfuric acid method, the incomplete reaction produces large amounts of residue, which also contain unreacted ilmenite. Large amounts of residue are generally accumulated without any treatment. Hydrocyclone use is regarded as a method for separating and recovering chemicals from process residues by which the unreacted components can be recycled efficiently. However, hydrocyclones designed by conventional procedures may have some limitations regarding classification sharpness. In this paper, numerical experiments and laboratory tests were performed to evaluate the classification sharpness of various hydrocyclone designs. Response surface methodology was used to optimize hydrocyclones with different structural configurations. Based on the response models, a designed hydrocyclone with a high sharpness of classification of particles was constructed. The sharpness of the newly designed hydrocyclone increased from 80.5% to 93.3%. The vortex finder separated approximately 89.9% of the fine particles in impurities, while 51.0% of TiO_2_ was recycled by the spigot. The hydrocyclone proposed in this paper properly minimizes the risk of environmental pollution caused by TiO_2_ production and provides a significant estimated cost savings.

## 1. Introduction

Titanium dioxide is widely used in coatings, rubber, plastic and everyday materials due to its superior whiteness, as well as its stable physical and chemical properties. The production of TiO_2_ by the sulfuric acid method produces a large amount of partial hydrolysis residues, consisting of either impurities or non-reactive raw materials and sulfuric acid [1]. The generation of waste is estimated at 30% of the total processed ilmenite when residues are untreated, and this waste is an environmental pollution risk that remains to be solved. Therefore, the separation and recovery of acid hydrolysis residue has great significance and needs to be addressed with urgency.

Currently, there are five main separation methods for acid hydrolysis residue recycling [2–4], comprising extraction, alkaline and acid leaching, flotation separation, magnetic separation and gravity separation. The first three processes mentioned require chemical treatments and inevitably generate secondary waste.

The essence of magnetic separation is to make use of the magnetic differences among mineral particles to separate the mixture under the action of magnetic force and mechanical force. Xin Du [5] tested the feasibility of separating acid hydrolysis residue by magnetic separation. Although the titanium grade of recovered minerals can reach 49%, the high impurity removal rate is at the expense of the loss of titanium concentrate, and the recovery rate of minerals is low (only 20%). The hydrocyclone is a representative device used to realize particle classification using centrifugal force. This device has many advantages, such as convenient operation, easy adjustment and control, stable performance, a simple structure, no moving parts, a low maintenance cost, a large handling capacity, a small volume and a small footprint. Therefore, hydrocyclone technology is considered to be a kind of physical separation with noteworthy aspects. It has been reported that hydrocyclones have been used to treat water [6], fly ash [7] and other waste [8].

The simple geometry and operation of hydrocyclones conceal a depth of knowledge regarding the fluid mechanism and the structural configurations that affect the separation performance significantly [9–11]. It has been shown that a lower underflow rate could be beneficial for accurate separation by increasing the overflow diameter or decreasing the underflow diameter. Other studies [12,13] have determined overflow diameters in an appropriate range to allow a high sharpness of particle classification to be achieved by reducing the percentage of misplaced particles. Wang *et al*. [14] reported that the reverse-flow cylinder length in hydrocyclones played a non-essential role in separation performance.

In addition, more methods have been applied to study the correlation between separation performance and hydrocyclone structure. Chu *et al*. [15] investigated the comprehensive effects of structural modifications on operating performance with the orthogonal design method. Silva *et al*. [16,17] optimized the structural configuration of hydrocyclones by using both the differential evolution technique and the multi-objective firefly colony algorithm. The hydrocyclone design was capable of providing both a high separation efficiency and low energy consumption. Slack *et al*. [18] developed an automated computational fluid dynamics (CFD) tool for hydrocyclone design.

This study evaluated the design of a hydrocyclone with high sharpness and the development of a simple and effective method for industrial application in TiO_2_ plants as a study case. The interactive effects of structural configuration on the achievement of a high sharpness of particle classification by hydrocyclones used in acid hydrolysis residue recycling were evaluated by central composite circumscribed design (CCD). The range of values for individual structural components for CCD was determined according to a previous work, and response models between the structural components and the classification sharpness were introduced. A high-efficiency hydrocyclone for residue recycling was constructed, and very good particle classification was successfully achieved.

## 2. Material and methods

### 2.1. Materials

**Figure 1.**
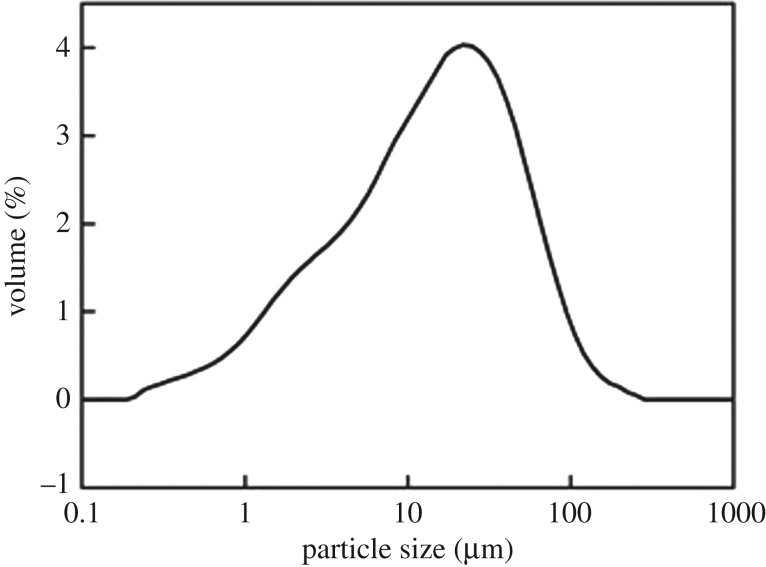
Particle size distribution of the acid hydrolysis residue.

**Figure 2.**
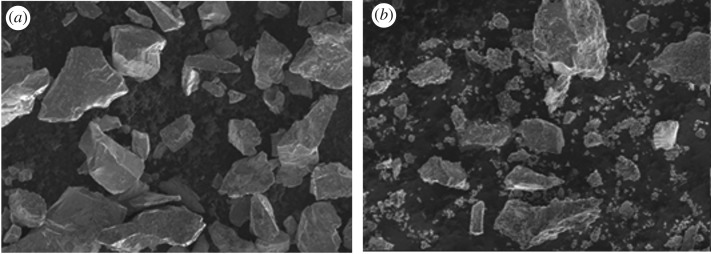
SEM images of ilmenite (*a*) and waste residue (*b*).

Residues discharged in the production of TiO_2_ by the sulfate method were used as raw materials. Figure 1 shows the particle size distribution, and table 1 provides the chemical compositions. Particles larger than 25 µm mainly contained ilmenite with 21% TiO_2_, and particles smaller than 25 µm were mainly silica sludge with 8% TiO_2_. Figure 2 shows SEM photos of the ilmenite and residue.

**Table 1.** Particle size distribution with different size by screening.

mesh	aperture (μm)	content (%)	density (g · cm^−3^)	TiO_2_%	cumulative TiO_2_ (%)	SiO_2_%	Fe_2_O_3_%
+120	+120	0.32	3.47	29.78	10.96	37.7	21.15
−120 + 140	+109–120	0.59	3.41				
−140 + 200	+75–109	1.33	3.43				
−200 + 240	+61–75	0.46	3.71				
−240 + 300	+48–61	4.56	3.80				
−300 + 400	+38–48	13.07	3.66	27.67	29.05	41.25	19.21
−400 + 500	+25–38	7.81	3.57	26.66	39.47	40.78	18.71
−500 + 800	+18–25	13.99	3.54	26.85	58.27	37.54	19.17
−800 + 1000	+13–18	55.42	2.96	14.56	98.66	51.71	9.95
−1000	−13	2.36	2.84	11.36	100.00	63.97	8.15

The geometry of the original hydrocyclone is presented in table 2. Table 3 shows the studied ranges of single parameters optimized in the previous work [13]. The four significant parameters were determined by CCD.

**Table 2.** Geometry of the original hydrocyclone.

parameter	symbol	dimension (mm)
diameter of the body	*D*_c_	75
diameter of inlet	*D*_i_	25 (same area quadrant is used)
diameter of vortex finder	*D*_o_	25
diameter of spigot	*D*_u_	12.5
length of vortex finder	*L*_o_	50
length of cylindrical part	*L*_c_	75
length of conical part	*L*	186

**Table 3.** Ranges and levels of the four dimensionless factors.

variable	symbol	range	+*β*	−*β*	+1	−1	0
*X*_1_	*D*_i_/*D*_c_	0.20 ∼ 0.30	0.30	0.20	0.28	0.23	0.25
*X*_2_	*D*_o_/*D*_c_	0.35 ∼ 0.60	0.60	0.35	0.54	0.42	0.48
*X*_3_	*L*/*D*_c_	1.25 ∼ 2.00	2.00	1.25	1.82	1.44	1.63
*X*_4_	*D*_u_/*D*_c_	0.05 ∼ 0.15	0.15	0.05	0.13	0.08	0.10

### 2.2. Mathematical design of CCD and index definition

As one of the most widely used procedures of response surface methodology, CCD was applied in the mathematical design. The independent input variables were the diameter of the vortex finder (*X*_1_), the diameter of the spigot (*X*_2_), the diameter of the inlet (*X*_3_) and the length of the cone (*X*_4_). The index of classification sharpness (*K*) was taken as the output response variable of the system. The ranges and levels of the four dimensionless factors are shown in table 3. Previous investigations were conducted to determine the extreme ranges of the variables. In this study, the total number of required tests was 30, including 16 factorial points, six centre points and eight axial points.

The four dimensionless factors were acquired from a series of tests (equation (2.1)), from which the coefficients were obtained by the least-squares method. Consequently, for the four variables, the response model was as follows:

2.1 $$Y=\beta_{0}+\sum_{i=1}^{4}\beta_{i}X_{i}+\sum_{i=1}^{4}\beta_{ii}X_{i}^{2}+\sum_{i<j}^{4}\beta_{ij}X_{i}X_{j}+\varepsilon ,$$

where *Y* is a response variable; *X_i_* and *X_j_* are the coded levels of the variables; *β_i_* is the regression coefficient for linear effects; *β_ii_* and *β_ij_* are the regression coefficients for quadratic effects; and ɛ is the error of prediction.

The mass flow rates of the two outlets were calculated by weighing timed samples. After the unit operations of filtration and drying, the concentrations of the samples were determined by measuring the mass of the solid phase. The size distributions of the particles were measured by a Malvern Mastersizer 2000 analyser. Then, the split ratio and the separation efficiency of the hydrocyclone were determined by the following equations:

2.2 $$S=\frac{Q_{u}}{Q_{i}}\times 100\text{\%}$$

and

2.3 $$G_{\left(i\right)}=\frac{M_{u}\times u_{i}}{M_{u}\times u_{i}+M_{o}\times v_{i}}\times 100\text{\%},$$

where *Q_u_* and *Q_i_* (kg s^−1^) are the flow rates of the slurry discharged from the spigot and the inlet, respectively; *M_u_* and *M_o_* (kg s^−1^) are the flow rates of the solid phase from the spigot and the vortex finder, respectively; and *u_i_* (%) and *v_i_* (%) are the volume fraction of particles that have a diameter of *i* from the spigot and the vortex finder, respectively.

The best limit of recovery was defined as a particle size of 25 µm; particles larger than this value were collected by the spigot, and smaller particles were collected by the vortex finder. Therefore, the sharpness index was defined as follows:

2.4 $$K=\frac{\left(E_{O}+E_{U}\right)}{2}.$$

where *E_O_* is the percentage of particles that are smaller than 25 µm, separated by the overflow, and *E_U_* is the percentage of particles that are larger than 25 µm, separated by the underflow.

**Figure 3.**
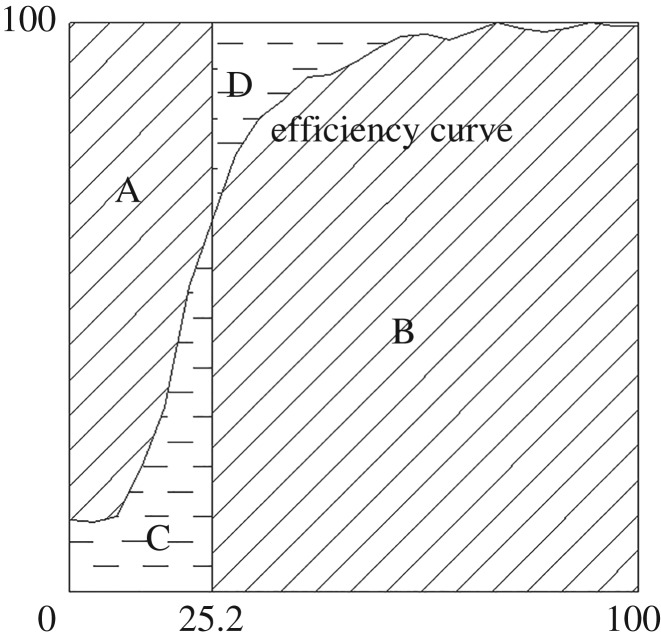
Schematic diagram of the index of classification sharpness.

Figure 3 shows a schematic diagram of the index of classification sharpness. In this study, both *E_O_* and *E_U_* are equally weighted in the equation of the sharpness index *K*.

### 2.3. CFD model description

The governing equations for the velocity field in an incompressible fluid can be written as

2.5 $$\frac{\partial \rho }{\partial t}+\frac{\partial p}{\partial x_{i}}(\rho u_{i})=0$$

and

2.6 $$\frac{\partial (\rho u_{i})}{\partial t}+\frac{\partial }{\partial x_{j}}(\rho u_{i}u_{j})=-\frac{\partial p}{\partial x_{i}}+\frac{\partial }{\partial x_{j}}\left[\mu \left(\frac{\partial u_{i}}{\partial x_{j}}+\frac{\partial u_{j}}{\partial x_{i}}\right)\right]+\frac{\partial }{\partial x_{j}}(-\rho \bar{u_{j}^{}u_{i}^{}}).$$

The velocity can be decomposed into its mean and fluctuating components,

2.7 $$u_{i}=\bar{u_{i}}+u_{i}^{\prime },$$

where $\bar{u_{i}}$ is the mean velocity; $u_{i}^{\prime }$ is the fluctuating velocity (*i* = 1, 2, 3); and $-\rho \bar{{u^{\prime }}_{i}{u^{\prime }}_{j}}$ is the Reynolds stress term including the turbulence closure, which must be modelled in order to close equation (2.6).

Therefore, the key component in the description of the fluid dynamics of hydrocyclones is the turbulence closure model, for which an appropriate turbulent model must be applied for the characterization of rotating turbulent flow. The Reynolds stress model (RSM) has been proved to predict anisotropic turbulence well, and this model has been selected to describe rotating turbulent flow in hydrocyclones. According to the RSM, $-\rho \bar{{u^{\prime }}_{i}{u^{\prime }}_{j}}$ is modelled by the following equation:

2.8 $$\frac{\partial }{\partial t}(\rho \bar{{u^{\prime }}_{i}{u^{\prime }}_{j}})+\frac{\partial }{\partial x_{k}}(\rho u_{k}\bar{{u^{\prime }}_{i}{u^{\prime }}_{j}})=D_{T,ij}+P_{ij}+\varphi_{ij}+\varepsilon_{ij},$$

where *ρ* is the liquid density; *u_i_* is the velocity; $u_{i}^{\prime }$ is the velocity fluctuation; *x_i_* is the positional length; $\partial /\partial t(\rho \bar{{u^{\prime }}_{i}{u^{\prime }}_{j}})$ is the local time derivative of the stress; and $\partial /\partial x_{k}(\rho u_{k}\bar{{u^{\prime }}_{i}{u^{\prime }}_{j}})$ is the convective transport term.

The interface between the liquid phase and air phase was simulated by the volume of fluid model (VOF), whose model equation is

2.9 $$\frac{\partial \alpha_{k}}{\partial t}+u_{j}\frac{\partial \alpha_{k}}{\partial x_{i}}=0,$$

where *α_k_* is the volume fraction of the *k*th phase, which varies between 0 and 1; and *u_j_* is the velocity component in direction *j*.

**Figure 4.**
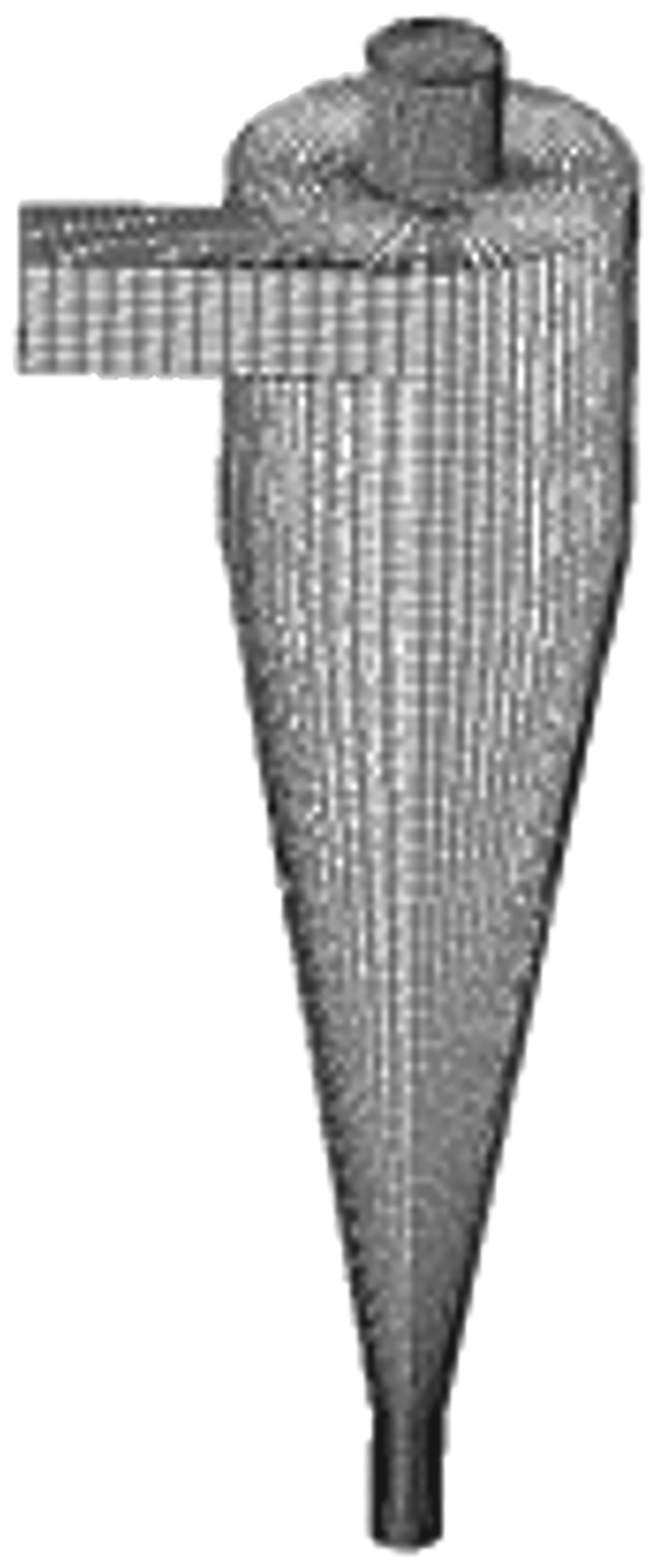
Grid representation of hydrocyclone.

Figure 4 shows the computational domain of the original hydrocyclone, which was divided into 259 000 unstructured hexahedral grids. The grids were refined near the walls and vortex finder. A grid independence test was conducted, and the validation result showed that 259 000 cells were optimal for balancing prediction accuracy and computational cost. The simulations were conducted using the ANSYS 16.2 software. Second-order upwinding and the SIMPLE pressure-velocity coupling algorithm were used. The convergence strategy used the unsteady solver, and the time step was chosen as 10^−4^–10^−3^ s. Trial tests showed that the results were not sensitive to the time step in this range. In this work, the time step was chosen as 5 × 10^−3^ s.

The hydrocyclone inlet was set as ‘velocity inlet’ boundary condition, and the velocities of liquid phase and solid phase were both 2.28 m s^−1^, because the differences on velocities of liquid and solid are few. In the real operations, the same velocities of liquid and solid phases were set. The ‘pressure outlet’ condition was applied to the outlets (vortex finder and spigot). The size distribution of particles was fitted by the Rosin–Rammler model. The main fitted parameters are spread parameter (1.26329) and number of diameters (25). Furthermore, the characteristic diameter was 19.85 µm, and the distribution coefficient was 1.26. The true density of the particles was 2057.9 kg m^−3^.

The CFD mathematical model was validated for numerical experiments by comparing the predicted velocity profiles with the experimental data at different axial locations. The experimental flow field was taken from Hsieh [19]. The predicted results of the velocity field were consistent with the experimental data. The grade efficiency of the particles was compared to the classification data, indicating a good qualitative agreement. These results validated the numerical models for extension to different configurations [13,20,21].

### 2.4. Experimental material and set-up

**Figure 5.**
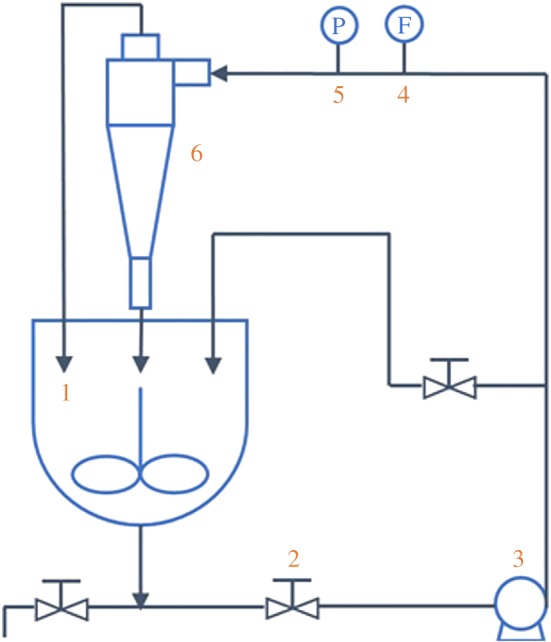
Experimental set-up of hydrocyclones for performance testing.

The classification of waste residues by the hydrocyclone was carried out in the laboratory. Figure 5 shows the experimental set-up, which consisted of a mortar pump, a mass flowmeter, a hydrocyclone and a tank. Waste residues and tap water were mixed in the tank to make up the suspension at a certain concentration (3.4 wt% in this study). The classification process can be summarized as follows: the slurry was pumped to the hydrocyclone at a certain flow rate, which was controlled by the sluice valve. The instantaneous value of the flow rate was displayed by the mass flowmeter. A constant flow rate was ensured to maintain good particle classification performance in the hydrocyclone. After accomplishing the classification process, the concentrated slurry of ilmenite was discharged into the tank from the underflow of the hydrocyclone. In addition, the slurry with silica sludge was discharged to the tank by the overflow. After the system reached stable conditions, sampling was performed at the two outlets, and the samples were analysed to determine the performance index of the classification.

### 2.5. Analysis method and processing of TiO_2_

Wet solid-phase screening tests were carried out for acid residues with different sieves of 120, 140, 200, 240, 300, 400, 500, 800 and 1000 mesh. The sieve residue and screen underflow were characterized after drying.

The acid hydrolysis residue was converted to a pulp with water. Then, the pulp was filtered and washed. The filtrate was mixed with deionized water in a 2000 ml volumetric flask to form a solution, and the chemical composition of the liquid phase was analysed by inductively coupled plasma atomic emission spectrometry (ICP-AES).

### 2.6. Model validation

**Figure 6.**
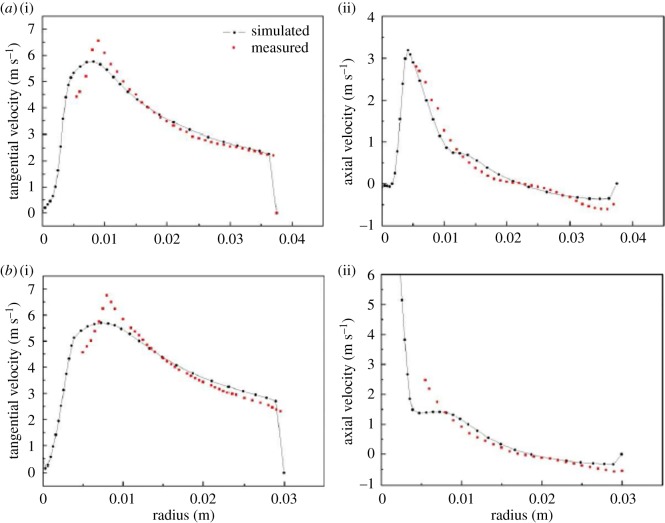
The comparison between the measured and simulated tangential (*a*(i),*b*(i)) and axial (*a*(ii),*b*(ii)) velocities at different locations from the top wall of a 75 mm hydrocyclone: (*a*) 60 mm and (*b*) 120 mm.

The mathematical model of CFD was validated for numerical experiments by comparing the predicted velocity profiles with the experimental data at different axial locations. The experimental flow field was taken from Hsieh [19]. As shown in figure 6, the predicted results of velocity field were consistent with the experimental data. The grade efficiency of the particles was compared to the classification data, indicating a good qualitative agreement. These works showed that the numerical models were validated to be capable of extending to different configurations.

## 3. Results and discussion

### 3.1. Design of a hydrocyclone with high sharpness

#### 3.1.1. CCD model and statistical analysis

The numerical results of the dimensionless factors for different structural components are summarized in table 4. A response model in coded units was obtained, which is shown as follows:

3.1 $$\begin{array}{rl} K=\; & 90.07-0.50X_{1}-2.87X_{2}+0.71X_{3}+0.058X_{4}-0.20X_{1}X_{2}\\ & +0.14X_{1}X_{3}+0.025X_{1}X_{4}+0.33X_{2}X_{3}+0.19X_{2}X_{4}\\ & -0.20X_{3}X_{4}-0.077X_{1}^{2}-0.56X_{2}^{2}-0.16X_{3}^{2}+0.073X_{4}^{2} \end{array}$$

where *K* is the response variable, and *X*_1_, *X*_2_, *X*_3_ and *X*_4_ are dimensionless factors.

**Table 4.** Simulated and predicted values of the dimensionless factors by the CCD model.

	coded level of variables				observed	observed	observed	predicted
test	*X_1_*	*X_2_*	*X_3_*	*X_4_*	*E_O_* (%)	*E_U_* (%)	*K* (%)	*K* (%)
1	−1	1	−1	−1	98.0	74.1	86.1	85.9
2	−1	−1	−1	−1	97.1	87.9	92.5	92.3
3	−1	1	1	−1	95.9	79.6	87.8	88.0
4	−1	−1	1	1	92.4	92.6	92.5	93.1
5	−1	−1	1	−1	96.1	90.9	93.5	93.1
6	−1	1	1	1	95.2	80.8	88.0	88.0
7	−1	−1	−1	1	96.1	89.3	92.7	92.3
8	−1	1	−1	1	96.9	75.7	86.3	85.9
9	1	1	1	−1	97.3	75.8	86.6	87.0
10	1	1	1	1	96.0	77.1	86.6	87.0
11	1	−1	1	−1	97.0	88.9	93.0	92.1
12	1	−1	−1	1	95.2	87.9	91.6	91.3
13	1	−1	1	1	93.2	90.8	92.0	92.1
14	1	−1	−1	−1	97.3	85.6	91.5	91.3
15	1	1	−1	1	97.3	72.3	84.8	84.9
16	1	1	−1	−1	97.9	69.9	83.9	84.9
17	−2	0	0	0	94.3	86.5	90.4	91.1
18	0	−2	0	0	90.2	95.2	92.7	93.6
19	0	0	−2	0	98.3	77.3	87.8	88.0
20	0	0	0	−2	97.7	82.0	89.9	90.1
21	2	0	0	0	98.0	80.1	89.1	89.1
22	0	2	0	0	97.3	68.4	82.9	82.1
23	0	0	2	0	95.6	86.4	91.0	90.8
24	0	0	0	2	96.5	85.1	90.8	90.1
25	0	0	0	0	96.7	82.9	89.8	90.1
26	0	0	0	0	96.7	82.9	89.8	90.1
27	0	0	0	0	97.5	82.9	90.2	90.1
28	0	0	0	0	97.5	82.9	90.2	90.1
29	0	0	0	0	97.5	82.9	90.2	90.1
30	0	0	0	0	97.5	82.9	90.2	90.1

The maximum relative error between the predicted *K* and the experimental values is approximately 3.7%, which is adequate precision to predict the classification sharpness for different hydrocyclone structures. Meanwhile, the correlation coefficient (*R*^2^ = 0.9856) shows that 98.56% of the variability in the response could be explained by the regression model. Adequate precision is determined by the signal to noise ratio, and it has been reported that a ratio greater than 4 is desirable. In this study, the ratio of 34 indicates an adequate signal. Thus, the response model could be used to optimize the hydrocyclone design for high sharpness.

Table 5 lists the analysis of variance (ANOVA) for the response model of the independent variables at the desired significance level, *p* < 0.05. As shown, *X*_1_, *X*_2_, *X*_3_, *X*_2_*X*_3_ and $X_{2}^{2}$ are significant variables at the 5% level. The other terms of *X*_4_, *X*_1_*X*_2_, *X*_1_*X*_3_, *X*_1_*X*_4_, *X*_2_*X*_4_, *X*_3_*X*_4_, $X_{1}^{2}$ and $X_{4}^{2}$, whose *p*-values are higher than 0.10, are not significant. It could be concluded that the significance of the structural components with respect to sharpness follows the order given here: the diameter of the vortex finder (*X*_1_) > the cone angle (*X*_4_) > the diameter of the inlet (*X*_3_) > the diameter of the spigot (*X*_2_). To refine the response model, the insignificant terms were removed. The modified response model is shown as follows:

3.2 $$\begin{array}{rl} K=\; & 90.06-0.50X_{1}-2.87X_{2}+0.71X_{3}\\ & +0.33X_{2}X_{3}-0.56X_{2}^{2}-0.16X_{3}^{2}. \end{array}$$

As a result, the maximum relative error of the modified response model is approximately 1.2%, indicating satisfactory agreement.

**Table 5.** Analysis of variance (ANOVA) for the response model.

source	sum of squares	d.f.	mean square	*F*-value	*p*-value	significance
model	228.90	14	16.35	73.28	<0.0001	***
*X*_1_	6.00	1	6.00	26.89	0.0001	***
*X*_2_	197.23	1	197.23	883.98	<0.0001	***
*X*_3_	12.04	1	12.04	53.97	<0.0001	***
*X*_4_	0.082	1	0.082	0.37	0.5542	*
*X*_1_*X*_2_	0.64	1	0.64	2.87	0.1110	*
*X*_1_*X*_3_	0.30	1	0.30	1.36	0.2624	*
*X*_1_*X*_4_	1.00 × 10^−02^	1	1.00 × 10^−02^	0.045	0.8352	*
*X*_2_*X*_3_	1.69	1	1.69	7.57	0.0148	***
*X*_2_*X*_4_	0.56	1	0.56	2.52	0.1332	*
*X*_3_*X*_4_	0.64	1	0.64	2.87	0.1110	*
$X_{1}^{2}$	0.16	1	0.16	0.73	0.4062	*
$X_{2}^{2}$	8.74	1	8.74	39.19	<0.0001	***
$X_{3}^{2}$	0.74	1	0.74	3.33	0.0880	**
$X_{4}^{2}$	0.15	1	0.15	0.65	0.4315	*
residual	3.35	15	0.22			
lack of fit	3.13	10	0.31			
pure error	0.21	5	0.043			
total	232.25	29				

#### 3.1.2. RSM plotting and optimization of structural configuration

**Figure 7.**
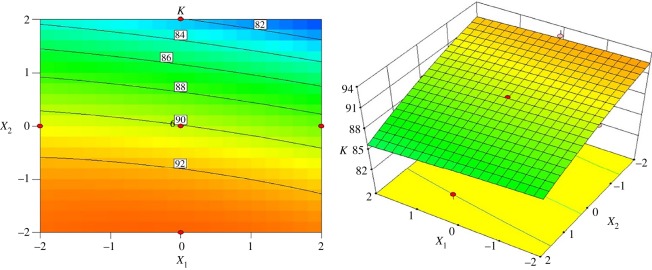
Contour and surface plots for the index *K* in coded values of *X*_1_ and *X*_2_.

To obtain a better understanding of classification performance within the hydrocyclone design, the interactive effects of structural components on the sharpness were compared through three-dimensional response surface plots. Figure 7 represents the interactive effects of the inlet diameter and vortex finder diameter on classification sharpness when the cone length is 122.25 mm and spigot diameter is 7.50 mm. When the vortex finder diameter is in the range of 26–32 mm, the inlet diameter has a minor effect on the index *K*, as indicated by the constant *K* value of 92%. Once the vortex finder diameter exceeds the value of 32 mm, an increase in the inlet diameter leads to a slight decrease in the index *K*. Notably, the index *K* decreases to 82% when the inlet diameter and vortex finder diameter simultaneously satisfy the ranges of 19–23 mm and 43–45 mm, respectively.

**Figure 8.**
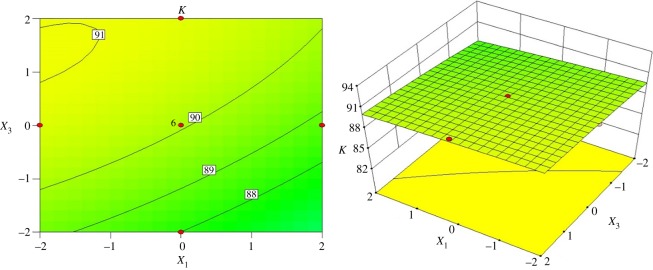
Contour and surface plots for the index *K* in coded values of *X*_1_ and *X*_3_.

Figure 8 shows the interactive effects of the inlet diameter and cone length on the index *K* when the vortex finder diameter is 36.00 mm and the spigot diameter is 7.50 mm. A high index *K* of 91% can be achieved when the inlet diameter and cone length are in the range of 15–17 mm and 133–147 mm, respectively. When the inlet diameter is kept constant, the index *K* will increase to 90% with increasing cone length. Once the inlet diameter is above 19 mm, the index *K* decreases to 88% when the cone length ranges from 94 to 105 mm.

**Figure 9.**
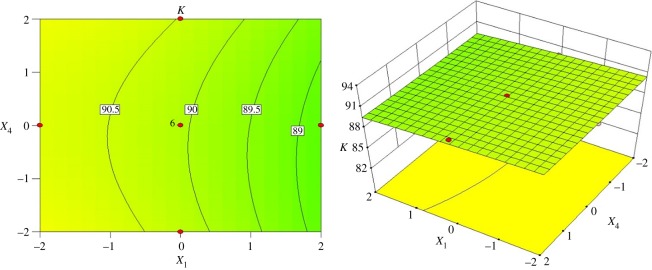
Contour and surface plots for the index *K* in coded values of *X*_1_ and *X*_4_.

Figure 9 shows the interactive effects of the inlet diameter and spigot diameter on the index *K* when the vortex finder diameter is 36.00 mm and the cone length is 122.25 mm. The index *K* remains almost the same in the studied range of spigot diameters, which shows the insignificance of spigot diameter with respect to the classification sharpness. When the inlet diameter is in the range of 15 mm to 19 mm, the index *K* is above 90%. By increasing the inlet diameter, the index *K* decreases from 91% to 89%.

**Figure 10.**
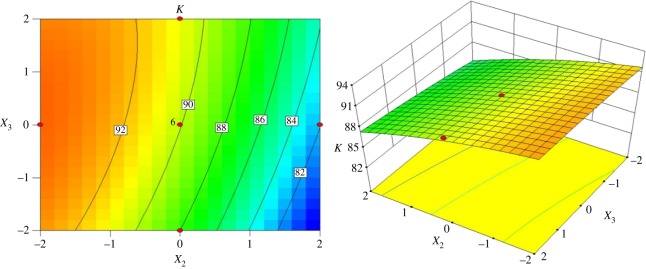
Contour and surface plots for the index *K* in coded values of *X*_2_ and *X*_3_.

The interactive effects of the vortex finder diameter and cone length on the index *K* are illustrated in figure 10 for an inlet diameter of 18.75 mm and spigot diameter of 7.50 mm. Regardless of cone length, a high index *K* of 92% can be achieved when vortex finder diameter is in the range of 26–28 mm. When the vortex finder diameter is above 28 mm, the index *K* slightly increases with an increase in the cone length. Then, the index *K* is lower than 82% when the vortex finder diameter and cone length range from 43 to 45 mm and 94 to 122 mm, respectively.

**Figure 11.**
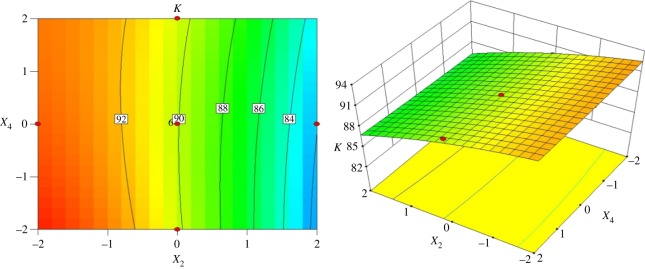
Contour and surface plots for the index *K* in coded values of *X*_2_ and *X*_4_.

The interactive effects of the vortex finder diameter and spigot diameter on the index *K* are shown in figure 11 when the inlet diameter is 18.75 mm and the cone length is 122.25 mm. The contour line is nearly vertical, which means that in comparison with the vortex finder diameter, the effect of spigot diameter on classification sharpness is weak. While maintaining the spigot diameter constant, the index *K* decreases from 92% to 84% when the vortex finder diameter is increased from 26.25 mm to 45 mm. The sharpness of classification remains at a high level when the vortex finder diameter ranges from 26 mm to 32 mm.

**Figure 12.**
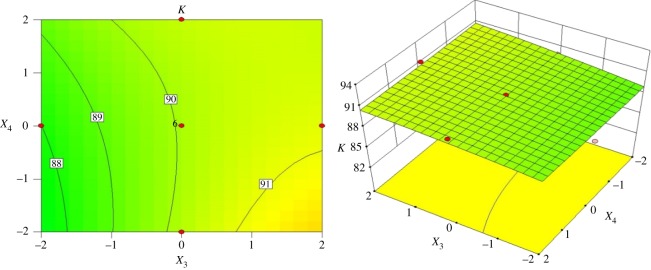
Contour and surface plots for the index *K* in coded values of *X*_3_ and *X*_4_.

The interactive effects of the cone length and spigot diameter on the index *K* are illustrated in figure 12 when the inlet diameter is 18.75 mm and the vortex finder diameter is 36.00 mm. The index *K* retains a high value of 91% when the cone length and spigot diameter are in the range of 133–150 mm and 4–6 mm, respectively. Throughout the full range of spigot diameters, the index *K* slightly increases with an increase in cone length. Finally, the index *K* decreases to 88% when the cone length and spigot diameter are in the range of 94–100 mm and 4–8 mm, respectively.

### 3.2. Comparison between the original and newly designed hydrocyclone for residue recycling

#### 3.2.1. Classification performance analysis of the original hydrocyclone

**Figure 13.**
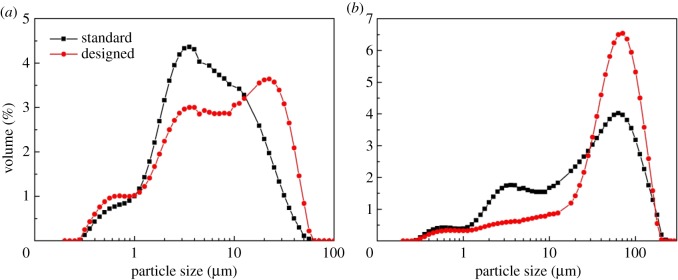
Comparison of the particle size distribution of the (*a*) overflow and (*b*) underflow samples for the original and designed hydrocyclones.

**Figure 14.**
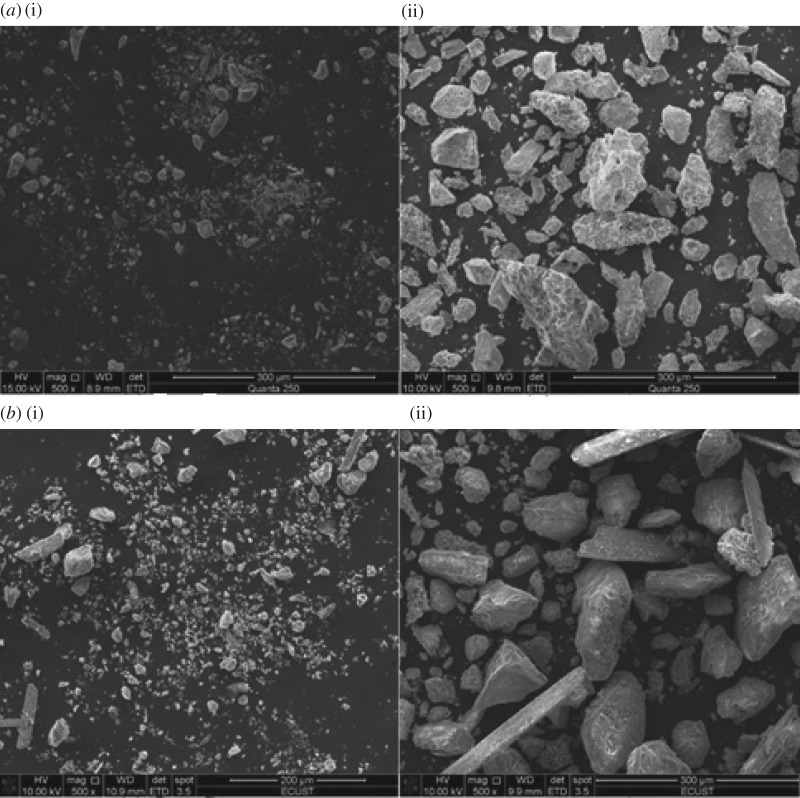
SEM images of the samples from the overflow (*a*(i),*b*(i)) and underflow (*a*(ii),*b*(ii)) of the original (*a*) and designed (*b*) hydrocyclones.

As shown in table 6, a new designed hydrocyclone was determined with the optimum conditions of 0.20 for *X*_1_, 0.35 for *X*_2_, 1.67 for *X*_3_ and 0.10 for *X*_4_, obtained through the above analysis. The classification performances of the original and designed hydrocyclones were evaluated experimentally. Figure 13 shows the difference in the size distribution of samples between the original and designed hydrocyclone. The fine and coarse particles of the acid hydrolysis residue were significantly classified by both the original and designed hydrocyclones, which indicated that the hydrocyclone can be regarded as a useful technology. Compared with the original hydrocyclone, the D[4,3] of the particles in the overflow and underflow of the designed hydrocyclone increased from 9.2 to 12.8 µm and 43.6 to 61.0 µm, respectively. This result indicates that the designed hydrocyclone could improve the classification of fine particles. Figure 14 shows SEM images of the samples from the overflow and underflow of both original and designed hydrocyclones. Differences can be observed in the particle size structures of the samples obtained from the overflow, which could be identified as sediment containing some silica. The underflow samples are mainly dense granules, which are the unreacted ilmenite. Furthermore, the proportion of fine particles in the underflow obtained from the designed hydrocyclone is lower than that in the underflow obtained from the original hydrocyclone.

**Table 6.** Geometry of the designed hydrocyclone.

parameter	symbol	dimension (mm)
diameter of inlet	*D*_i_	15 (same area quadrant is used)
diameter of vortex finder	*D*_o_	26.25
diameter of spigot	*D*_u_	7.5
length of conical part	*L*	125

**Figure 15.**
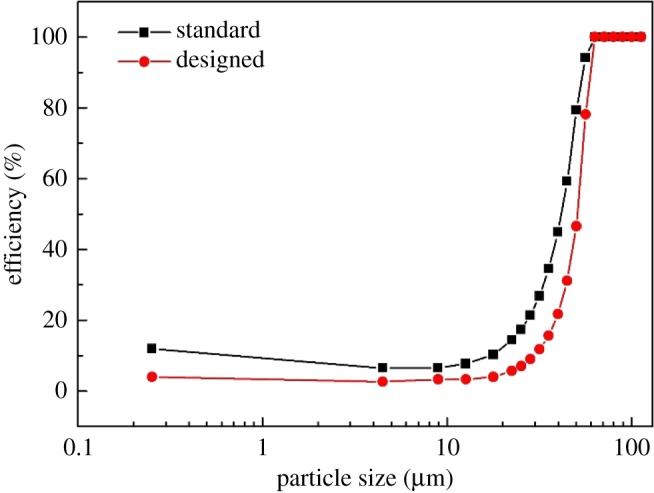
Separation efficiency curves of the original and designed hydrocyclones.

A comparison of the grade efficiency curves is illustrated in figure 15, and the detailed results in table 7 show that the vortex finder separated 89.9% of the fine particles in impurities and that 51.0% of the TiO_2_ was recycled by the spigot. In this case, a high sharpness classification of waste residues was achieved by the designed hydrocyclone.

**Table 7.** Sharpness indexes of the original and designed hydrocyclones.

hydrocyclone	*E_O_*_­_ (%)	*E_U_* (%)	*K* (%)
original	67.0	93.9	80.5
designed	89.9	96.7	93.3

**Figure 16.**
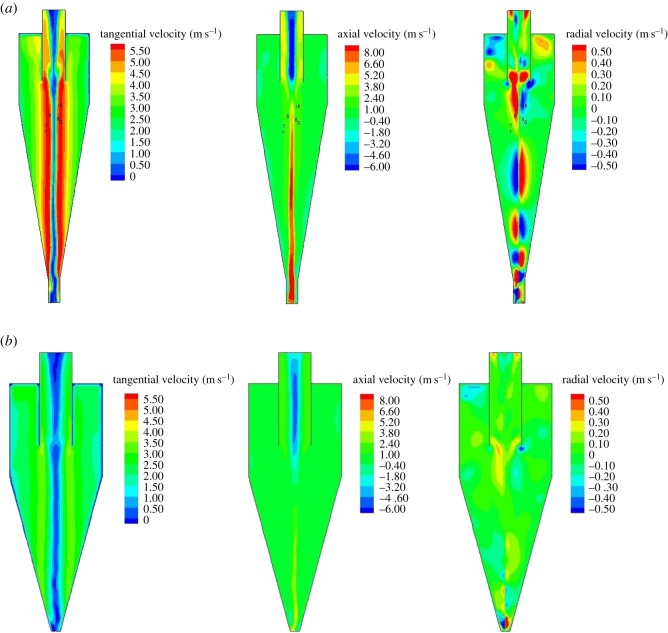
Description of the fluid flow in the original (*a*) and designed (*b*) hydrocyclones.

**Figure 17.**
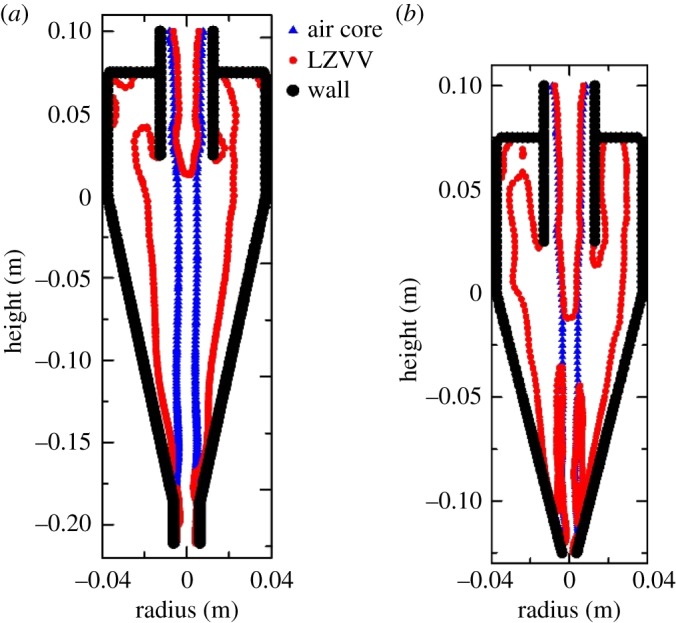
Air core and LZVV in the original (*a*) and designed (*b*) hydrocyclones.

The velocity distributions in the original and designed hydrocyclones are illustrated in figure 16. Comparing the velocity gradient, the designed hydrocyclone shows a smaller value, with a maximum tangential velocity of approximately 3–4 m s^−1^ and axial velocity of −2 to 2 m s^−1^. The difference in the axial velocity between these two hydrocyclones has an effect on the shape of the locus of zero vertical velocity (LZVV). The particles inside the LZVV flow from the overflow, whereas those outside the LZVV flow from the underflow. The properties of the air core in the middle of the hydrocyclone affect the performance of the hydrocyclone. Even though there are no particles in the air core, the space between the air core and the LZVV directly determines the fine-particle separation efficiency. As shown in figure 17, the space in the designed hydrocyclone is larger than that in the original one, which means that it is easier for fine particles to be discharged from the vortex finder.

## 4. Conclusion

A design study of hydrocyclones with structural configurations evaluated by response surface methodology and numerical analysis was successfully performed, and the optimized parameters were evaluated in pilot-scale experiments, the principal results of which are summarized as follows:

(1) Response surface methodology is a highly efficient way to address multi-parameter processes. High sharpness has been predicted to classify acid hydrolysis residues and recycle the unreacted ilmenite successfully. The vortex finder separated 89.9% of the fine particles in impurities, and 51.0% of TiO_2_ was recycled by the spigot. The results obtained in the designed hydrocyclone prove the high classification-sharpness efficiency of these residues.
(2) Four dimensionless factors were proposed in the CCD model of the hydrocyclone, and the effects of these parameters on the sharpness follow the order given here: the diameter of the vortex finder (*X*_2_) > the length of the cone (*X*_3_) > the diameter of the inlet (*X*_1_) > the diameter of the spigot (*X*_4_).
(3) Acid hydrolysis residue could be recycled through hydrocyclones with high efficiency, and approximately US$1 million per year could be saved, according to the actual industrial production process. The high-value elements could be reused, and the pollution problem could be solved by a single piece of equipment.

## Data accessibility

Data are deposited at Dryad Digital Repository: https://doi.org/10.5061/dryad.57kk82c [22].
